# Barriers and facilitators to delivering tobacco cessation interventions at HIV care clinics in Sub-Saharan Africa: Qualitative application of the consolidated framework for implementation research

**DOI:** 10.18332/tpc/221806

**Published:** 2026-07-30

**Authors:** Kyra Guy, Jennifer Tsui, Ezekiel Musasizi, Ronald Kusolo, Musawa Mukupa, Jim Arinaitwe, Fastone Goma, Lynn Atuyambe, David Guwatudde, Masauso M. Phiri, Cosmas Zyambo, Heather Wipfli

**Affiliations:** 1 Department of Population and Public Health SciencesUniversity of Southern CaliforniaLos AngelesUnited States; 2 School of Public HealthMakerere UniversityKampalaUganda; 3 Centre for Primary Care ResearchUniversity of ZambiaLusakaZambia; 4 School of Public HealthUniversity of MarylandCollege ParkUnited States

**Keywords:** cessation, global health, prevention, low/middle income countries, priority/special populations

## Abstract

**Introduction:**

Tobacco use among people living with HIV (PLWH) in Sub-Saharan Africa (SSA) is prevalent, compounding health risks. Tobacco cessation interventions (TCIs) have shown promise but are not widely tested in HIV care settings. In this study, we applied the Consolidated Framework for Implementation Research (CFIR) to examine barriers and facilitators to implementing the adapted tobacco cessation intervention Quit4life+ for PLWH in Uganda and Zambia.

**Methods:**

Guided by the CFIR determinant framework, Quit4life+ trial implementation challenges were evaluated across 16 HIV clinics in Uganda and Zambia between July 2021 and October 2025. Qualitative data were drawn from multiple sources, including 22 quarterly site visit reports, 4 health systems assessments completed by study coordinators and health facility personnel in-charge, key informant interviews with 14 clinic healthcare providers (nurses, nursing officers, and midwives), and focus group discussions with 55 PLWH (aged ≥18 years and receiving HIV care at participating clinics). Qualitative data were mapped to relevant domains and constructs.

**Results:**

Within the innovation domain, evidence for TCIs, nicotine replacement therapy (NRT), and culturally adapted message libraries enhanced acceptability, while costs challenged adoption. Outer setting challenges, including seasonal labor demands, civil unrest, poor road access, and restrictive procurement and regulatory processes, influenced delivery. Inner setting barriers included overcrowded clinics, limited counseling space, inconsistent network connectivity, and staff turnover. Providers were motivated to support cessation but reported limited skills and faced intersecting stigma among patients. The implementation process relied on external resources, raising concerns about sustainability.

**Conclusions:**

Findings identify multilevel barriers and facilitators to delivering cessation support within HIV care clinics in Uganda and Zambia. Further research is needed to determine how determinants can be effectively addressed and to build the evidence base for the sustainable integration of services into HIV care across SSA.

## Introduction

Increased access to antiretroviral therapies (ART) has significantly improved life expectancy among people living with HIV (PLWH)^1^. Despite this progress, behavioral risk factors, including tobacco use, continue to threaten overall health outcomes^2^. With nearly one in four deaths among PLWH on ART being attributable to tobacco smoking, tobacco use is a leading contributor to excess morbidity and mortality among PLWH^3^. Across Sub-Saharan Africa (SSA), PLWH are disproportionately more likely to use tobacco than their HIV-negative counterparts, with prevalence being approximately one and a half times higher among men and nearly twice as high among women^4^. For example, in Uganda, the prevalence of smoking among PLWH is 20% for men and 6% for women, compared with 10% and 2% for general population men and women^4^. In this population, tobacco use accelerates HIV disease progression, reduces ART adherence, and heightens vulnerability to opportunistic infections such as tuberculosis; these physical risks are further compounded by elevated rates of depression and psychological distress, which are themselves associated with greater tobacco use among PLWH^5,6^.

In response to this significant risk, Article 14 of the World Health Organization Framework Convention on Tobacco Control (WHO FCTC) calls for the development and implementation of effective measures to promote tobacco cessation interventions (TCIs) and adequate treatment for tobacco use dependence^7^. However, little research has been conducted on the use of these TCIs for PLWH in the SSA region^8^. A recent systematic review conducted by Peer et al.^9^ in SSA identified only eight studies in SSA, all conducted in South Africa^9^. These included five randomized controlled trials (RCTs), two quasi-experimental studies, and one case-control study^9^. The interventions tested various cessation methods, including pharmacotherapy, behavior counseling, or a combination of both, but only one study specifically focused on PLWH^9^. Additional research is needed to understand what constitutes effective interventions for this population. PLWH face distinct structural and resource challenges in the region, including higher tobacco use rates, unique barriers to quitting, and increased health risks^10^.

To address this need, we adapted and tested a multi-component tobacco cessation intervention for PLWH, known as Quit4Life+, in Uganda and Zambia^11^. The intervention comprised cessation strategies including standard clinician-delivered brief advice, text message support, and NRT. In addition to assessing the relative effectiveness of the different strategies on cessation at six months, we also examined factors influencing the implementation of the Quit4life+ components in the study context. Guided by the widely used Consolidated Framework for Implementation Research (CFIR), this study aimed to assess barriers and facilitators influencing the delivery and sustainability of cessation interventions across five domains (innovation, outer setting, inner setting, individuals, and implementation process)^12^. This article presents key qualitative findings from the study to provide insight on the potential scale-up of tobacco cessation programming for PLWH across SSA.

## Methods

### Study setting and design

Qualitative implementation-related data were collected from research staff, clinic health workers, and PLWH involved in the delivery of the multi-phased Quit4Life+ intervention between July 2021 and September 2025. Quit4Life+ was carried out across 16 HIV care clinics from two districts in Uganda and two districts in Zambia. Selected districts in Uganda included Arua, located in the agrarian West Nile region, and Moroto, located in the nomadic northeastern region. In Zambia, the selected districts were Mongu in the Western Province and Chipata in the Eastern Province. Health service delivery in both countries is structured across a tiered system ranging from community-based health centers (II-IV) to district, regional, and national referral hospitals. HIV care in both countries is highly decentralized and primarily delivered free of charge through local health centers^13^.

**Figure 1:**
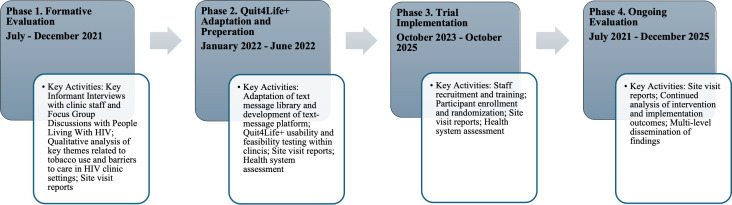
Study design, implementation phases, and timeline for the Quit4life+ intervention and data collection in HIV clinics in Uganda and Zambia, 2021–2025 (N=800)

Delivery of the intervention was done in four phases (Figure 1). Prior to implementation of the clinical trial, phase one consisted of formative research, including focus group discussions (FGDs) and key informant interviews (KIIs) with PLWH who use tobacco and HIV clinic health service workers. This was followed by an adaption phase in which the Quit4Life+ Short Message Service (SMS) program, grounded in the World Health Organization-International Telecommunication Union (WHO-ITU) *Be He@lthy, Be Mobile Framework,* was tailored to the cultural and contextual realities of the study context^13^. Adaptations included expanding the text message library to address both combustible and non-combustible tobacco use, incorporating context-specific health risks of tobacco use, and modifying message tone and language. The full adaptation methodology is reported in detail elsewhere^13^.

A randomized controlled trial was then implemented in which 800 PLWH who use tobacco in Uganda and Zambia were enrolled and randomized to one of the following four treatment arms: 1) standard of care; 2) standard of care + provision of NRT; 3) standard of care + Quit4Life + text-messaging; and 4) standard of care + NRT + Quit4life + text-messaging. The primary outcome of interest was tobacco use cessation by study participants 6 months post-enrollment. Further information on the trial’s design and full study protocol have been previously published^14^.

### Qualitative data collection and management

Multiple data sources were reviewed throughout the Quit4Life+ implementation process to examine experiences relevant to the five principal CFIR domains (Table 1). Given CFIR’s extensive use in implementation research and its demonstrated applicability in SSA contexts, it served as an appropriate and relevant framework to guide the collection, organization, and analyses of the presented qualitative data^15,16^.

**Table 1:** Qualitative data sources, data collection procedures, and corresponding participants for the evaluation of the Quit4life+ intervention in HIV clinics in Uganda and Zambia, 2021–2025

Source	Data collection methods	Participants
**Site visit reports** (N=22)	Study staff visited the 16 participating clinics pre-implementation and quarterly through the study phases to monitor progress and document logistical or operational challenges. Field reports were completed by in-charges at health facilities using a standardized tool with checklist variables evaluating site performance based loosely on Consolidated Framework for Implementation Research (CFIR) constructs.	Uganda and Zambia study coordinators; Health facility personnel in-charge
**Health system assessments** (N=4)	Structured health systems assessments were completed in 2022 and 2025 (one assessment per country) to evaluate organizational readiness for implementation. Assessments were based on inner setting CFIR constructs and examined clinic infrastructure, staffing, training, workflow, and current service delivery capacity.	Uganda and Zambia study coordinators; Health facility personnel in-charge
**Key informant interviews** (N=14)	Key informant interviews (KIIs) explored provider perspectives on barriers and facilitators to delivering tobacco cessation support within HIV clinics. Eligible providers were identified by facility leadership. Interviews lasted~1 h, followed a standardized guide, and were conducted privately by trained research staff in the local language. All sessions were audio-recorded and transcribed.	Clinic healthcare providers, including nurses (n=9), midwives (n=1), nursing officers (n=4), primarily female (78%), mean age ≈ 41 years
**Focus group discussions** (N=55)	Eight focus group discussions (FGDs) (6–8 participants each) were conducted with PLWH to explore experiences, acceptability, and perceived barriers related to cessation support. Sessions lasted~1 h, followed a standardized guide, and were privately conducted by trained research staff in the local language. All sessions were audio-recorded and transcribed.	PLWH aged ≥18 years receiving HIV care; mean ages 40–47 years; majority male in most districts; primarily married and unemployed across sites

Site visit reports (n=22) and standardized health systems assessments (n=4) were completed by study coordinators to document ongoing challenges in each study country. Additionally, KIIs (n=14) with clinic health workers and FGDs (n=55) with PLWH were conducted to explore more in-depth perspectives on barriers and facilitators to delivering and engaging with tobacco cessation support services within the selected HIV clinics^17^.

Study materials were securely stored on access-restricted servers managed by the research team and all data were de-identified to ensure confidentiality. Written informed consent was obtained from all participants at each data collection point. Ethical approval for all study procedures was granted by the University of Southern California, the Makerere University School of Public Health in Uganda, the Uganda National Council for Science and Technology, the University of Zambia Biomedical Research Ethics Committee, and the Zambian National Health Research Ethics Committee.

### Data analysis

#### Identification of implementation factors

Qualitative data analysis followed an iterative, multi-stage process guided by the updated CFIR framework. The study principal investigator (HW) and country-level coordinators (KG, EM, MM) first conducted an initial review of all documentary data sources (Table 1). Preliminary themes were identified inductively from transcripts, focusing on emerging implementation experiences related to intervention delivery, operational challenges, barriers, and facilitators. Data were coded after interpretation and analysis using DeDoose Software V.9.054^18^. Two study investigators (KG, EM) independently coded a subset of transcripts, compared coding decisions, and refined initial themes. A third investigator (HW) reviewed discrepancies until consensus was reached.

Selected implementation themes (experiences) were reviewed during weekly virtual multisite meetings involving the full research teams from Uganda, Zambia, and the US. These meetings served to collaboratively validate experiences, discuss interpretation, and address any challenges. Detailed meeting notes were maintained and shared with study partners and relevant stakeholders, if necessary, for further confirmation and refinement.

#### Mapping of CFIR domains and constructs

Using a deductive qualitative approach, the identified implementation themes were subsequently mapped to the publicly available CFIR domains and associated constructs^13^. Factors mapped within the domains and constructs were further categorized as a barrier or facilitator to Quit4Life+ implementation. Qualitative analysis continued until thematic saturation was reached. Criteria for thematic saturation were defined under the following criteria: 1) no new CFIR constructs were identified after coding consecutive data sources, 2) no new barriers or facilitators emerged within previously identified domains/constructs, and 3) the same constructs were identified across multiple sources. The final analytic structure was discussed with the study team, adjusting to ensure accuracy and consistency across countries:

Through this process, the CFIR domains were operationalized for this study as the following: the innovation domain encompassed features of the text-message program, NRT delivery, and costs; the inner setting described organizational context and capacity within HIV clinics; the outer setting captured patient needs, community conditions, and external policy environments; the individuals domain reflected patient and provider attitudes; and the implementation process examined intervention planning, coordination, and execution. Results for selected domains and related constructs are presented visually, and with illustrative examples and supporting quotations.

## Results

Qualitative data were drawn from 22 site visit reports and 4 health systems assessments completed by study coordinators and clinic personnel in-charge across 16 HIV clinics. Additional KIIs with 14 clinic healthcare providers (nurses, nursing officers, and midwives; 78% female; mean age 41 years), and 8 FGDs with 55 PLWH aged ≥18 years and receiving HIV care (ages 40–47 years; majority male; primarily married and unemployed) were also completed.

**Figure 2:**
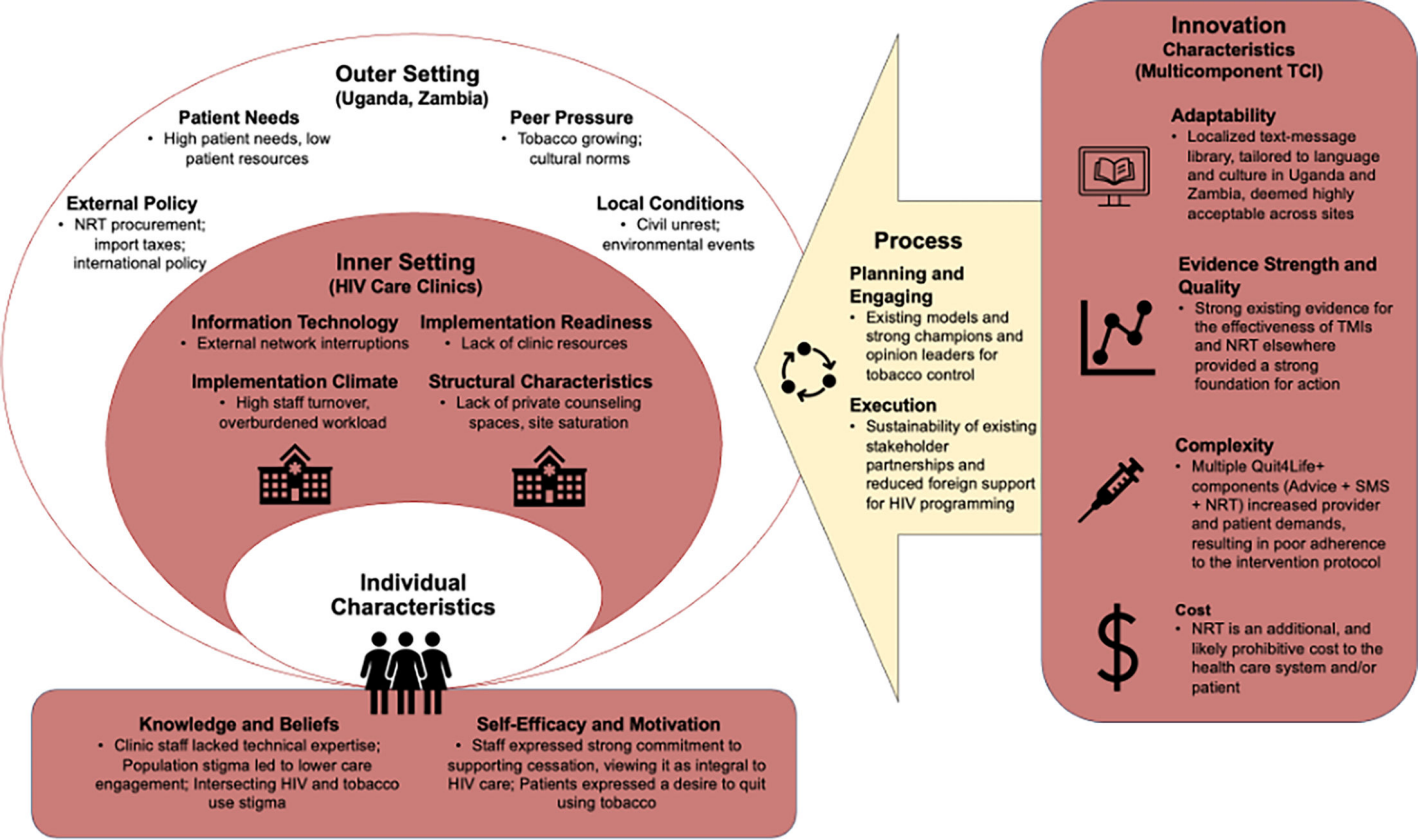
Visualization of CFIR domains and related constructs for the implementation of the Quit4Life+ intervention in HIV clinics in Uganda and Zambia, 2021–2025

Of the 48 constructs and subconstructs assessed within the CFIR domains, 19 were classified as either barriers, facilitators, or both to the delivery of the Quit4life+ intervention within HIV clinics in Uganda and Zambia (Figure 2). Illustrative examples and supporting quotations of selected CFIR domains and constructs are presented in Table 2.

**Table 2:** Examples for the implementation of the Quit4Life+ intervention in HIV clinics in Uganda and Zambia, 2021–2025

CFIR domain	Construct	Illustrative examples	
		Barrier	Facilitator
**Innovation characteristics**	Adaptability		Localized text-message library, tailored to language and culture in Uganda and Zambia, deemed highly acceptable across sites
	Evidence strength and quality		Strong existing evidence for the effectiveness of text message interventions (TMIs) and nicotine replacement therapy (NRT) elsewhere provided a strong foundation for action
	Design quality		Previously adapted and tested design in low- and middle-income countries (LMICs) based on WHO’s *Be He@lthy, Be Mobile* initiative improved program quality
	Complexity	Multiple Quit4Life+components (advice+text-messages + NRT) increased provider and patient demands, resulting in poor adherence to the intervention protocol	
	Cost	NRT is an additional, and likely prohibitive cost to the health care system and/or patient	Evidence shows that TMIs have been cost-effective as behavior change interventions in both countries
**Outer setting**	Patient needs and resources	High patient needs and low resources within the population result in the prioritization of issues other than tobacco cessation	
	Peer pressure	Tobacco growing in some areas and cultural norms promoting tobacco use resulted in poor outcomes	
	External policies and laws	NRT is not domestically available in either country, complicating procurement and distribution Import taxes and communication-authority (UCC) approval processes International and local policy changes through the implementation period Shifts in broader HIV service delivery models	
	Local conditions	Civil unrest in Karamoja limited site visits for supervision Adverse environmental events (farming season patterns, flooding/drought) Unreliable national network infrastructure limiting timely text-message delivery	
**Inner setting**	Information technology infrastructure	External network interruptions disrupted clinic workflows, including verification of TMI delivery and timely data synchronization	
	Structural characteristics	Overcrowded HIV clinics lacked private counseling areas, leading to rushed or public conversations about cessation Poor road infrastructure in remote clinics limited access for implementation support Site saturation, certain remote clinics with few tobacco-using patients required staff to search for patients, slowing recruitment	
	Implementation climate	High staff turnover required repeated training; new staff often lacked orientation to Quit4Life+ Providers prioritized antiretroviral therapy-related tasks over cessation due to overburdened workload	
	Readiness for implementation	Limited availability of clinic staff for consistent training and implementation Lack of clinic resources	In-charges and other trained clinic staff helped with encouraging implementation and maintaining consistent coordination
**Individuals**	Knowledge and beliefs about intervention	Staff lacked technical expertise in delivering brief advice to quit tobacco and administer NRT Population stigma led to lower engagement by health care providers Intersecting HIV and tobacco use stigma negatively impacts patients’ care-seeking behavior	
	Self-efficacy/ motivation		Staff expressed strong commitment to supporting cessation, viewing it as integral to HIV care Patients expressed a desire to quit using tobacco
**Implementation process**	Planning		Existing models available to adapt and implement intervention in local contexts, including formative research guides and training materials
	Engaging		Existence of strong champions and opinion leaders pushing for tobacco control Strong stakeholder engagement [local HIV and non-communicable disease (NCD) actors, HIV clinics, policymakers]
	Execution	Sustainability of existing partnerships between NCD and HIV teams is required Reduced foreign support for HIV-related health programming	
	Reflecting and evaluating	Ongoing monitoring and coordination of intervention delivery required weekly team meetings and check-ins	Multisite weekly meetings enabled rapid problem-solving (e.g. addressing connectivity failures)

CFIR: Consolidated Framework for Implementation Research.

### Innovation

Within the innovation domain, referring to the combined use of text messaging, brief advice to quit, and NRT to support cessation among PLWH, more facilitators than barriers were identified. Strong evidence base, previous application in other contexts, and adaptability to local conditions were reported as facilitators of implementation. Providers viewed Quit4Life+ text-messaging as a credible, evidence-based intervention aligned with WHO recommendations and feasible within clinics to support PLWH in quitting tobacco:

*‘I think the SMS is a good strategy because it constantly reminds the tobacco users not to smoke or to not use tobacco. It’s a good encouragement. These texts create an environment similar to that of the health Centre.’* (Clinic Staff, Uganda)

The contextual adaptability of the Quit4Life+ message library emerged as another strong innovation characteristic facilitator. Adaptations made through the formative study phase were seen to address prominent cultural differences across study regions (e.g. variations in tobacco use behaviors, limited community education on health risks, and strong social acceptance of tobacco as a part of cultural norms), enhancing patient comprehension. Clinic patients reported enjoying tailored text message content and language that reflected specific population needs in terms of use, education, and literacy:

*‘A useful example of text is the one that talks about you trying to estimate the amount you spend on tobacco products every day in a year; it could be a lot. Like a member said, we don’t have financial education, which makes us use whatever money we have in a reckless manner.’* (Patient, Uganda)

*‘To my understanding, the second text is quite good. It explains to us how certain disease conditions are attributable to tobacco use and thus we should quit using tobacco to stay free from such diseases.’* (Patient, Zambia)

Further adaptations included program modifications that successfully increased participant engagement. The purchasing of solar chargers so mobile phones were able to continue receiving Quit4life+ messages, choices in the types and frequency of text messages, and dosage of NRT based on SSA context, were determined through the formative study phase.

In terms of barriers to the innovation domain, intervention costs presented as a consistent concern, with participants identifying distinct cost categories across program components. The text-messaging component was widely viewed as cost-effective for patients and scalable, but required centralized investment from funders to telecommunication companies to offset costs to patients. NRT introduced more substantial and recurring costs, including international procurement, import taxes, and regulatory approvals, none of which had clear domestic financing mechanisms. Clinics consistently expressed uncertainty about sustaining NRT without external support, identifying it as a pressing cost-related threat to long-term program viability.

### Outer setting

Within the outer setting, or the communities in which the intervention was implemented, several contextual barriers influenced Quit4Life+ delivery. Seasonal farming cycles, drought, and intermittent civil unrest limited both patient attendance and staff mobility. For example, clinic staff reported that participants were more difficult to follow up during peak farming seasons, especially in Uganda:

*‘Being near the border [Karamoja region of Uganda], some clients come from the neighboring countries, districts within the region, and from the refugee camps or settlements occasioned by insecurity, and as such are hard to follow-up.’* (Quarterly Site Visit Report 2022)

*‘The challenges experienced by the research assistants and the study participants include issues related to the farming season. During the farming season, the participants travel to work in the fields and have minimal opportunity to return to their homes from where to access health care.’* (Quarterly Site Visit Report 2023)

Poor road infrastructure in more remote clinics (Moroto and Chipata) also made routine travel for study maintenance and participant follow-up more difficult. Unreliable national telecommunications networks also caused periodic disruptions in text-message delivery, though operational consequences were felt most acutely at the inner-setting level.

Several other outer-setting challenges were attributed to country-level policy. Differences in HIV service delivery models between Uganda and Zambia introduced challenges to standardizing Quit4Life+ implementation across countries. For example, in Uganda, many patients visited clinics only once every 3–6 months for ART refills, making recruitment, NRT prescription, and dose-monitoring difficult to align with routine HIV care. In terms of NRT delivery, procurement in both countries required approval through multiple administrative channels, resulting in delays. For text messaging, SMS launch also required clearance from national communication authorities, which changed over the study period and stalled implementation timelines:

‘*Uganda Communications Commission (UCC) policy change: all those with more than 100 phones are now required to seek clearance from them directly, as opposed to service providers. All non-operational phones must be shut off.’* (Quarterly Site Visit Report 2025)

At an international level, unforeseen policy and external funding changes, including administrative changes from the United States’ National Institutes of Health (NIH) and Agency for International Development (USAID), created budgetary challenges and inconsistencies for both funders and local HIV clinics participating in the program:

*‘The recent stop-work orders issued by the U.S. government have significantly disrupted healthcare service delivery in Uganda and Zambia. In Uganda, the Ministry of Health (MoH) has requested affected health workers to volunteer for three months (February–April). However, this situation has led to widespread demotivation among staff, impacting their ability to report to work and effectively engage in research activities.’* (Quarterly Site Visit Report 2025)

### Inner setting

Within the inner setting, or the clinics in which the intervention was implemented, several operational barriers emerged. A central challenge involved information-technology infrastructure. Although national network reliability was within the outer setting domain, disruptions directly interfered with clinic workflows in the inner setting. Study site visits reported difficulty confirming message delivery, syncing data, and maintaining consistent communication with patients when connectivity was weak.

For clinic staff, physical infrastructure challenges, including overcrowded HIV clinics and limited private counseling space, constrained the ability to provide brief advice or discuss tobacco use confidentially. Staff described rushing through cessation counseling or having to conduct discussions in public areas.

Staffing constraints also shaped implementation. Many clinics experienced high turnover, requiring repeated training to orient new personnel to Quit4Life+. Heavy HIV workloads meant that cessation tasks were also often reported as being deprioritized:

*‘I think we need priority topics to talk and discuss with our clients, we don’t give enough time. This could be because we don’t see tobacco use as a priority.’* (Clinic Staff, Uganda)

In several rural clinics, site saturation also hindered recruitment. Certain study regions (Karamoja) had smaller numbers of PLWH who use tobacco, slowing recruitment and requiring staff to add sites and spend additional time identifying eligible participants:

*‘Some facilities have already screened all the participants, enrolled all eligible PLWHs, and followed them up to completion.’* (Quarterly Site Visit Report 2024)

### Individuals

At the individual level, both barriers and facilitators emerged among providers and patients. While staff were highly experienced in HIV care, many reported limited confidence in delivering structured cessation counseling or managing NRT prescriptions:

*‘Most of us have never had any form of training on tobacco use. So, sometimes it is hard to give the right information to the client. So, I think all these health workers should be empowered with knowledge about tobacco use.’* (Clinic Staff, Zambia)

Intersecting stigma related to HIV and tobacco use also affected provider–patient interactions. Some providers noted that patients feared being judged for smoking while participating in the intervention:

*‘We also experience tobacco stigma because we know that people do not like the odor that comes off us.’* (Patient, Uganda)

Despite these barriers, providers expressed a strong commitment to helping patients quit. Many framed tobacco use as a critical threat to treatment adherence and patient longevity:

*‘If all general healthcare workers in their training are taken through cessation modules, it would be wonderful ... but for now, it’s not there.’* (Clinic Staff, Uganda)

Study staff and clinicians also shared that their patients expressed a desire to quit, looking for easy and effective solutions within the clinics.

### Implementation process

With regard to the implementation process domain, extensive formative research and pilot testing were viewed as instrumental facilitators in initial success. The high level of preparation through training workshops, message-library validation, and early stakeholder consultations helped clarify roles and establish standard context-specific procedures. Although these experiences were seen as facilitators, participants expressed that continued engagement would likely be difficult without upfront external resource investment.

Similarly, while the Quit4life+ process was well structured and collaborative, its success relied heavily on external coordination, resources, and planning that were largely identified as barriers to sustainability. The intervention required multisectoral collaboration between the HIV and non-communicable disease sectors that are often disconnected and at times competitive with each other. These relationships expedited clinic buy-in and improved coordination, but there were recurring concerns about whether stakeholders would continue to invest in or develop policy for tobacco cessation, given increasing resource competition and shifting local and national priorities:

*‘The gap with tobacco use cessation is that there is no operational guidance from MoH at the moment.’* (Clinic Staff, Uganda)

*‘We do not have NRT listed as an essential drug. The stakeholders need to recognize it as an essential drug and then procure it through the National Medical Stores to the facilities.’* (Clinic Staff, Uganda)

Regular reflection and evaluation through site visits and weekly meetings among Ugandan, Zambian, and US partners were also important as successful constructs in maintaining program quality. Despite the success, this demanded considerable time and staff commitment in overburdened clinic environments.

## Discussion

Framed around the five CFIR domains, this study reported on barriers and facilitators of the Quit4Life+ tobacco cessation intervention for PLWH in Uganda and Zambia. Substantially more barriers to implementation were reported across the five domains, including: complexity and cost of delivering a multi-component intervention, overburdened HIV clinics, structural resource limitations, country-level policy changes, adverse environmental events, and fragility of local partnerships. Despite these contextual challenges, Quit4Life+ proved to be a feasible and acceptable model for delivering tobacco cessation support within HIV clinics. Several facilitators were identified, including the cultural adaptability of the localized text-message library, strong stakeholder engagement, routine monitoring, and motivation among clinic staff and patients.

As previously mentioned, the development of the Quit4Life+ SMS intervention was informed by the *Be He@lthy, Be Mobile* framework, which provided standardized guidance for the implementation of *mHealth* interventions across diverse settings^11^. CFIR findings suggest that grounding the intervention in an established evidence base was central to its usability and acceptability and informed key stages of intervention design^19^. For example, the formative research phase, described in detail in previous publications, enabled context-specific cultural adaptations to the text-message library. Both PLWH participants and clinic health workers viewed these features as integral to the overall acceptability and usability of the intervention’s text-message component^12^. Prior evaluations of text-message programs have reinforced these findings, suggesting that, particularly in LMICs, such iterative strategies to message wording and translation often enhance user comprehension and engagement^20^.

Like other applications of comprehensive *mHealth* programs, Quit4Life+ was designed to provide scalable technical support compatible with existing HIV service platforms^21^. Individual motivation was identified as a facilitator of this compatibility, with providers reporting that integration of Quit4Life+ into routine HIV care aligned with their goals of delivering holistic, patient-centered services. Providers perceived a relative advantage of this care approach when compared to traditional vertically oriented programs and praised the availability of NRT as a practical cessation approach for patients within study clinics^22^. The Quit4Life+ protocol was described as feasible within existing workflows, and many noted that it facilitated the initiation of tobacco cessation conversations that would not have otherwise occurred. In many clinics, this practice helped to establish a baseline standard of care for addressing tobacco use, reflecting similar successes reported by other efforts to leverage existing care platforms in expanding chronic disease prevention and control^23^. Clinic patients also praised the compatibility of the intervention, expressing interest in learning more about the health impacts of tobacco use and ways to engage in cessation programming. This inherent self-efficacy and motivation to quit have been consistent in SSA, with evidence suggesting that HIV positive smokers are receptive to quitting smoking^24^.

Despite encouraging usability feedback from providers and patients, many clinic-level barriers impacted implementation. As commonly cited structural challenges faced by HIV clinics in the region, staffing shortages, high patient volumes, limited counseling space, and a lack of formal tobacco cessation training hindered intervention fidelity^25,26^. The multicomponent nature of the intervention was further complicated by local conditions often present in communities throughout SSA, including civil unrest and adverse weather events (e.g. farming seasons, flooding, drought). Adding cessation-related tasks to already strained environments risks exacerbating provider burden and research ability, demonstrating the need for dedicated training, workflow adaptation, and broader structural monitoring and investment^5,27^. It is also important to note that participating HIV clinics encountered constraints related to both inner setting technology connectivity and outer setting telecommunications networks, which are essential to the delivery of text-based behavioral change interventions. Similar challenges have been documented across other *mHealth* interventions in SSA, where unreliable network coverage and poor healthcare infrastructure have disrupted scalability^28^.

Stakeholder engagement emerged as another influential determinant of implementation. Early and continuous involvement of HIV clinic leadership, Ministry of Health actors, local research partners, noncommunicable disease (NCD) actors, and tobacco control stakeholders was said to have facilitated buy-in, aligned implementation priorities, and improved coordination across sectors. While seen largely as a facilitator, this level of involvement raised concerns related to sustainability. The Quit4Life+ program relied heavily on external financial and technical support, leading study participants to express uncertainty about whether tobacco control would receive investment beyond the study period. Especially given that this was a multi-country project, views of sustainability were also shaped by differing national policy environments^29^. For example, providers in both study countries consistently pointed to the absence of NRT on national essential medicines lists, noting that this policy change would significantly reduce implementation barriers and strengthen cessation care for PLWH. Unlike countries such as South Africa, where comprehensive tobacco control legislation, excise tax increases, and integration of cessation services into primary care have advanced over the past two decades, many countries in SSA, including Uganda and Zambia, continue to face gaps^30,31^. Based on these findings, we suggest that Ministries of Health in Uganda and Zambia work to add NRT to national formularies and provide local funding for cessation support and counseling within existing HIV care platforms. Broader implementation science literature reinforces these suggestions, emphasizing that sustained delivery in low-resource settings depends on multilevel solutions including coordinated policy advocacy, domestic financing mechanisms, and structural reform beyond externally supported project cycles^28,29,32^.

### Limitations

This qualitative study was conducted with several limitations in mind. First, although the qualitative sample (14 KIIs, 8 FGDs) was modest, it was purposively selected to support deductive CFIR mapping rather than inductive theory generation. Triangulation with 22 site visit reports and 4 health systems assessments across 16 clinics strengthened credibility, though low tobacco-use prevalence in some regions limited recruitment diversity at those sites.

Second, the qualitative data collected in this study were self-reported and therefore subject to recall and social desirability biases, which may have influenced how participants described barriers and facilitators to implementation.

Finally, although data were collected across diverse clinics in Uganda and Zambia, findings may not be fully generalizable to all HIV service delivery settings in SSA. Furthermore, while the CFIR provided a useful structure for identifying implementation determinants, its application may not capture all relevant contextual nuances of the rapidly shifting health system throughout the Quit4Life+ implementation period^33,34^. However, study findings offer meaningful insight into the tobacco cessation and HIV care environment across much of Uganda and Zambia and may inform efforts in environments with similar structural and operational conditions.

## Conclusions

Using the Consolidated Framework for Implementation Research, this study identified several multilevel determinants influencing the implementation of the Quit4Life+ intervention among PLWH in Uganda and Zambia. While Quit4Life+ demonstrated strong potential and was viewed as acceptable and feasible by both providers and patients, findings suggest that strengthening cessation support delivery within the realities of HIV care platforms throughout SSA requires further research and attention to factors beyond intervention design alone.

Generally, Quit4life+ implementation experiences identified through this study demonstrate that the long-term feasibility of integrating tobacco cessation support services into HIV care in SSA depends on broader health system readiness, and national and global policy alignment. Changing policy priorities and recent shifts in global funding especially highlight the vulnerability of short-term externally supported cessation programs for PLWH. In this context, sustained progress will require Ministries of Health and other stakeholders throughout the region to take deliberate, coordinated action: expanding prescription NRT availability, embedding stigma reduction and capacity building directly into cessation service workflows, and investing in the digital, structural, and stakeholder infrastructure necessary to support long-term integration within HIV care platforms. Without this foundation, programs like Quit4Life+ cannot achieve the scale or sustainability needed to meaningfully reduce disease burden among PLWH across SSA.

## Abbreviations

ART: anti-retroviral therapy
PLWH: People Living with HIV
SSA: sub-Saharan Africa
WHO: World Health Organization
TCIs: Tobacco Cessation Interventions
RCTs: Randomized Controlled Trials
NRT: Nicotine Replacement Therapy
WHO: World Health Organization
ITU: International Telecommunication Union
SMS: Short Message Service
CFIR: Consolidated Framework for Implementation Research
LMIC: Low- and Middle- Income Countries
KII: Key Informant Interview
FGD: Focus Group Discussion
UCC: Uganda Communications Commission
NIH: National Institutes of Health
USAID: US Agency for International Development
NCDs: Noncommunicable Diseases
